# Loratadine, an H_1_ Antihistamine, Inhibits Melanogenesis in Human Melanocytes

**DOI:** 10.1155/2019/5971546

**Published:** 2019-03-17

**Authors:** Hye-Rim Moon, Soo Youn Jo, Hak Tae Kim, Woo Jin Lee, Chong Hyun Won, Mi Woo Lee, Jee Ho Choi, Sung Eun Chang

**Affiliations:** ^1^Department of Dermatology, Korea University Ansan Hospital, Korea University, 123, Jeokgeum-ro, Danwon-gu, Ansan-si, Gyeonggi-do 15355, Republic of Korea; ^2^Asan Institute for Life Science, Asan Medical Center, University of Ulsan College of Medicine, 88 Olympic-ro 43-gil, Songpa-gu, Seoul 05505, Republic of Korea; ^3^Department of Dermatology, Asan Medical Center, University of Ulsan College of Medicine, 88 Olympic-ro 43-gil, Songpa-gu, Seoul 05505, Republic of Korea

## Abstract

It has long been believed that histamine is associated with cutaneous melanogenesis. Specifically, H2-receptor antagonists reportedly inhibit melanogenesis, but H1-receptor antagonists, which are some of the most commonly prescribed medicines in dermatology, have not been studied to determine whether and how they regulate melanogenesis. Therefore, we screened H1-receptor antagonists to determine whether they inhibit melanogenesis and found that loratadine was particularly effective, in this regard without compromising cellular viability. Loratadine downregulated microphthalmia-associated transcription factor (MITF) and tyrosinase in melanocytes. To determine the intracellular signaling pathways, Akt was consistently activated by loratadine. PI3K/Akt pathway inhibitor, LY294002, restored the reduced melanin content that was induced by loratadine. In addition, phospho-GSK-3*β* also was found to be increased following loratadine treatment. Loratadine reduced the amount of PKC-*β*II in the membrane fraction, thereby decreasing its activity. Taken together, our data indicate that loratadine regulates melanogenesis via Akt/MITF and PKC-*β*II signaling, thereby leading to the inhibition of melanogenic proteins. The antimelanogenic effects of loratadine have potentially significant and useful roles in dermatologic practice, although further clinical studies will be required to test this.

## 1. Introduction

Hyperpigmentary skin disorders are a major issue of concern for people with dark skin. Until now, clinicians have attempted to treat hyperpigmentary disorders using various therapeutic modalities, including hypopigmenting agents, but the results have been discouraging.

Diverse types of immune cells have been thought to play an important role in melanogenesis. Various inflammatory dermatoses can induce postinflammatory hyperpigmentation by affecting the morphologic change and functional activity of melanocyte. We recently reported the inflammatory features of melasma in Korean women and that mast cells are frequently found in the dermis of melasma lesions [1]. In addition, we proposed the association between increased dermal mast cells and clinical features of hyperpigmentation and telangiectasia in photodamaging process [2]. Therefore, inflammatory mediators such as histamine, prostaglandins, and nitric oxide have been identified as possible therapeutic approaches for hyperpigmentary skin disorders [3].

Histamine is a ubiquitously distributed inflammatory mediator that is released from tissue mast cells and blood basophils, thereby inducing various inflammatory skin disorders. Since the cutaneous actions of histamine as a melanogen were first reported [4], there have been studies on the role of histamine in skin hyperpigmentation [3, 5, 6]. Histamine-induced morphologic changes in human melanocytes and increased tyrosinase activity, resulting in increased melanogenesis and H_2_ antihistamines specifically, suppressed the stimulatory effects of histamine [5, 6]. However, the involvement of histamine in melanogenesis via other receptors such as H_1_, H_3_, and H_4_ receptors remains unclear. Especially considering common use of H_1_ antihistamines in dermatologic practice, it is remarkable that little has been reported about the effects of H_1_ antihistamines on melanogenesis. Therefore, we tried to focus for defining the effects of H_1_ antihistamines on melanogenesis.

## 2. Materials and Methods

### 2.1. Materials

Loratadine, 3,4-dihydroxy-L-phenylalanine (L-DOPA), cholera toxin (CT), 12-O-tetradecanoylphorbol-13-acetate (TPA), and antibodies specific to *β*-actin were purchased from Sigma-Aldrich Co. (St. Louis, MO, USA). Antibody specific for Akt (#2920S), phosphor-Akt (ser473, #4058S), *β*-catenin (#9562L), GSK-3*β* (27C10, #9315), phospho-GSK-3*β* (Ser9, #9336), p44/42 MAPK (Erk1/2) (#9102S), and p44/42 MAPK (Erk1/2) (Thr202/Tyr204, #9101S) were purchased from Cell Signaling Technology (Beverly, MA, USA). Antibodies specific for tyrosinase (C-19) and PKC-*β*II (C-18) were purchased from Santa Cruz Biotechnology, Inc. (Santa Cruz, CA, USA). Microphthalmia (MITF) Ab-1 (C5, MS-771-P0) was obtained from Neomarkers (Fremont, CA, USA).

### 2.2. Cell Culture

Normal human melanocytes (NHM) were obtained from Invitrogen and maintained in Medium 254 (Cascade Biologics, Portland, OR, USA), which contained human melanocyte growth supplement at 37°C in a 5% CO_2_ incubation [7]. The Mel-Ab cell line is a mouse-derived, spontaneously immortalized melanocyte cell line that synthesizes large quantities of melanin. Mel-Ab cells were maintained in Dulbecco's modified Eagle's medium (DMEM) supplemented with 10% fetal bovine serum (FBS), 100 nM TPA, 1 nM CT, 1% antibiotic-antimycotic solution (100X) at 37°C in 5% CO_2_. B16F10 cells were maintained in DMEM supplemented with 10% FBS and 1% antibiotic-antimycotic solution (100X) at 37°C in 5% CO_2_ [8].

### 2.3. Cell Viability Assay

Cell viability of NHM and Mel-Ab cells was measured using MTT (3-(4,5-dimethylthiazol-2-yl)-2,5-diphenyl tetrazolium bromide; Duchefa, Netherlands) assay. NHM and Mel-Ab cells were seeded at the same density in 24-well plate. After incubation with loratadine for 24 hr. MTT was prepared as 2.5 mg/ml stock solution in phosphate buffered saline (PBS) and stored at 4°C. Then, stock MTT solution was added at 200 *μ*l/well and the plates incubated at 37°C for 30 min to 4 hr. Thereafter, MTT solution was removed. After addition of 3 ml of DMSO the plates were incubated for 15 min at 37°C to dissolve the formazan crystals. Absorbance was measured at 570 nm using an enzyme immunosorbent assay (ELISA) reader (Molecular Devices Co., Sunnyvale, CA). The reference wavelength was 560-650 nm.

### 2.4. Melanin Contents and Microscopy

NHM and Mel-Ab cells were treated with specific agonists and for 5 days or 3 days. Cells were dissolved in 550 *μ*l of 1 N NaOH at 100°C for 30 min and centrifuged at 13,000 rpm for 5 min. The optical density (OD) of the supernatants was measured at 405 nm using a microplate reader. Before measuring the melanin content, the cells were observed under a phase contrast and photographed for microscope (Olympus, Tokyo, Japan).

### 2.5. Tyrosinase Activity

NHM and Mel-Ab cells were seeded in 6-well plates and incubated with loratadine for 5 days or 3 days. The cells were washed with ice-cold PBS and lysed with phosphate buffer (pH 6.8) containing 1% Triton X-100. The cells were then disrupted by freezing and thawing, and the lysates were clarified by centrifugation at 15000 rpm for 10 minutes. After quantifying the protein levels of the lysate and adjusting the protein concentrations with lysis buffer, 90 *μ*l of each lysate containing the same amount of protein was placed in each well of a 96-well plate, and 10 *μ*l of 10 mM L-DOPA was then added to each well. The control wells contained 90 *μ*l of lysis buffer and 10 *μ*l of 10 mM L-DOPA. Following incubation at 37°C, absorbance was measured every 10 min for at least 1 hr at 475 nm using a microplate reader.

### 2.6. Cell Fractionation

We used a cell fractionation kit from cell signaling. Cell fractionation was performed according to the manufacturer's instructions.

### 2.7. Western Blot Analysis

Cells were lysed in protein lysis buffer and centrifuged at 13,000 rpm for 30 min. The protein concentration was determined using a Bradford protein assay. 20 *μ*g of protein per lane was separated by SDS-polyacrylamide gel electrophoresis and blotted onto nitrocellulose membranes, which were then saturated with 5% skim milk in Tris-buffered saline containing 0.5% Tween 20. Blots were incubated with the appropriate primary antibodies following the manufacturer's data sheet. Image analysis was used to determine the relative band densities, which was performed using Image J software (https://imagej.nih.gov/ij/).

### 2.8. Total RNA Extraction and cDNA Synthesis

Total cellular RNA was extracted from the NHM using the Favor Prep™ Total RNA purification mini according to the manufacturer's instructions (Favorgen, Ping Tung, Taiwan). Following isolation, the quantity and quality of the RNA were determined using a NanoDrop ND-1000 spectrophotometer (ND-1000, NanoDrop Technologies, Inc. Wilmington, DE, USA). Single-stranded complementary DNA (cDNA) was synthesized from 1 *μ*g of total RNA using RevertAid First Strand cDNA Synthesis kit according to the manufacturer's instructions (Thermo Scientific, Rockford, IL, USA).

### 2.9. Real-Time RT-PCR

qRT-PCR was performed using the LightCycler® 480II machine coupled with SYBR Green chemistry (Roche Applied Science, Indianapolis, IN, USA). In terms of qRT-PCR settings, initial denaturation was performed at 95°C for 5 min, followed by amplification at 95°C for 10 sec, 60°C for 10 sec, and 72°C for 10 sec for 45 cycles. The cDNA obtained was amplified with the following primers: RPLP0, Forward 5′-GGCGACCTGGAAGTCCAACT-3′, Reverse 5′-CCATCAGCACCACAGCCTTC-3′. MITF, Forward 5′-ACTTTCCCTTATCCCATCCACC-3′, Reverse 5′-TGAGATCCAGAGTTGTCGTACA-3′. Primers specific for RPLP0 were used for loading control amplifications.

### 2.10. Statistics

The statistical significance of the differences between groups was assessed using analysis of variance (ANOVA), followed by the Student's* t*-test. In this study,* p* <0.05 is considered significant.

## 3. Results

### 3.1. Loratadine, an H1- Receptor Antagonist, Suppresses Melanogenesis in NHM and Mel-Ab Cells

**Figure 1:**
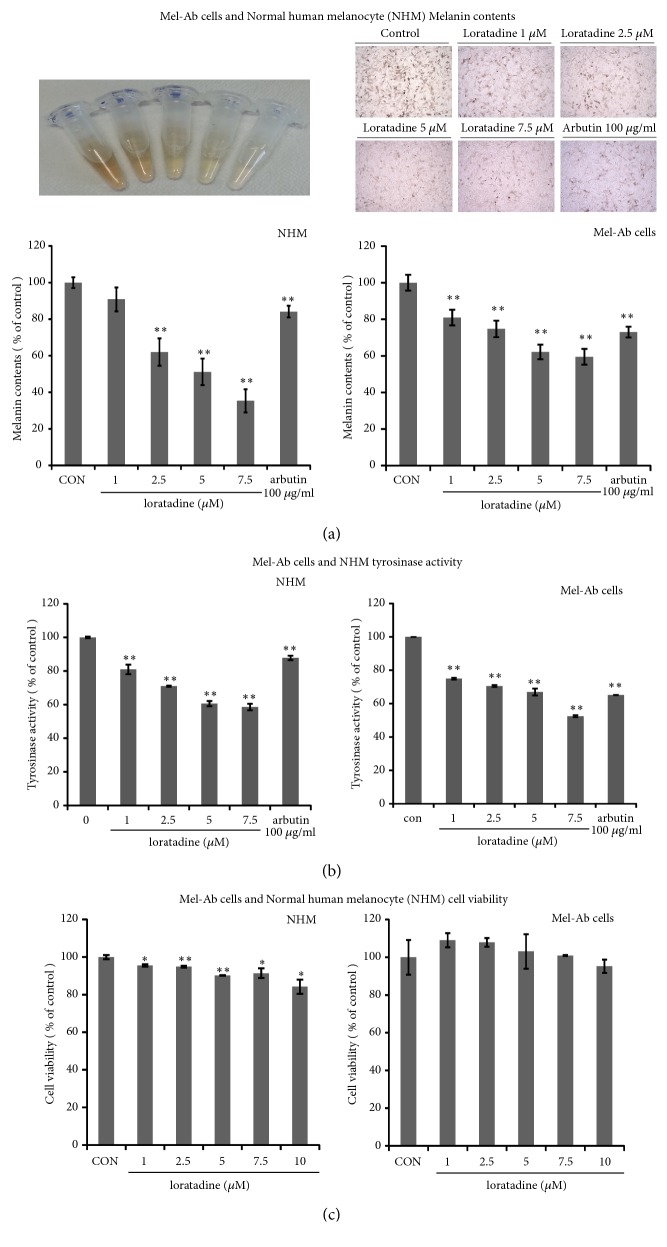
Effects of H_1_ antihistamine, loratadine, on melanogenesis in normal human melanocytes (NHM) and Mel-Ab cells. (a) NHM and Mel-Ab cells were cultured with 1.0-7.5 *μ*M loratadine for 5 days and 4 days each and compared with 100 *μ*g/ml arbutin. The cell images were taken using phase contrast microscopy, and the decrease in melanin is evident. (b) Loratadine suppressed tyrosinase activity in NHM and Mel-Ab cells. NHM and Mel-Ab cells were incubated with 1.0-7.5 *μ*M loratadine for 5 days and 4 days each, and the cellular tyrosinase activity was then measured. Arbutin (100 *μ*g/ml) was used as a positive control. Each determination was made in triplicate, and the data represent the mean ± SD. ^*∗*^p < 0.05 and ^*∗∗*^p < 0.01 in comparison with the untreated control. (c) The viability of NHM and Mel-Ab cells was not affected by treatment with 1.0-7.5 *μ*M loratadine for 24 hr. Cell viability was determined using MTT assay. Each measurement was made in triplicate.

Even though H2-receptor agonists and antagonists have been extensively studied previously, the effect of H1-receptor antagonists on melanogenesis has not been fully understood. First, we explored whether H1-receptor antagonists influenced the melanogenesis in B16F10 cells. Among the H1-receptor antagonists screened, ebastine, clemisole, terfenadine, and loratadine significantly decreased the melanin content (Table 1 and Supplementary Fig.  1A). We selected ebastine and loratadine as they decreased the melanin content in a dose-dependent manner. While ebastine affected cellular viability in NHM and Mel-Ab cells (data was not shown), loratadine showed a dose-dependent response without affecting cellular viability in NHM and Mel-Ab cells (Figures 1(a) and 1(c)). Also, loratadine treatment decreased the tyrosinase activity in a dose-dependent manner (Figure 1(b)).

To determine the involvement of H1-receptor agonist on melanogenesis, we evaluated the effects of 2-pyridylethylamine, an H1-receptor agonist. The H1 receptor agonist did not affect the melanin content in Mel-Ab cells or reverse the loratadine induced melanin reduction (Supplementary Figure 1B and 1C).

### 3.2. Loratadine Inhibits Expression Levels of MITF in NHM

**Figure 2:**
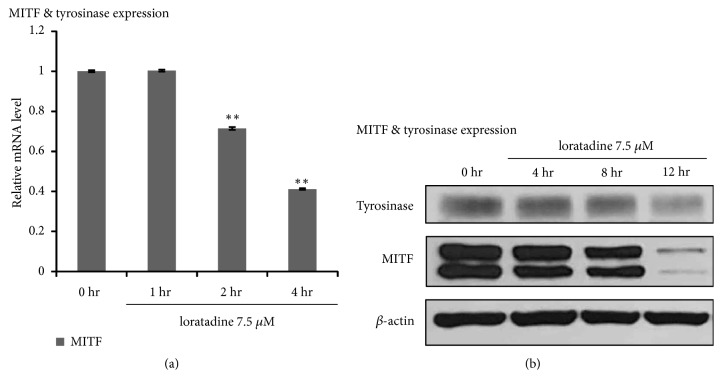
Inhibitory effect of loratadine on mRNA and protein expression of MITF and tyrosinase. (a) Loratadine dramatically decreased the mRNA levels of MITF after 4 hr. (b) Loratadine reduced the protein levels of MITF and tyrosinase. NHM were incubated with 7.5 *μ*M loratadine at indicated time. Whole-cell lysates were then analyzed by western blotting using antibodies against MITF and tyrosinase. Normalization was achieved by dividing the values for individual bands by the densitometry values for *β*-actin in the same lane. The value of densitometry was graphed with the mean ± SD. ^*∗*^p < 0.05.

As loratadine decreased melanin synthesis and tyrosinase activity, we next determined whether loratadine affected the expression of MITF, which plays crucial role in tyrosinase gene expression in melanogenesis. Loratadine significantly decreased the protein level of MITF and tyrosinase at 12 hr (Figure 2(a)). Furthermore, MITF and tyrosinase mRNA level also decreased after loratadine treatment (Figure 2(b)). These results indicated that loratadine reduced melanogenesis through the downregulation of MITF signaling pathway.

### 3.3. Inhibitory Effects of Loratadine on Melanogenesis Were Partially Associated with Akt Phosphorylation in NHM

**Figure 3:**
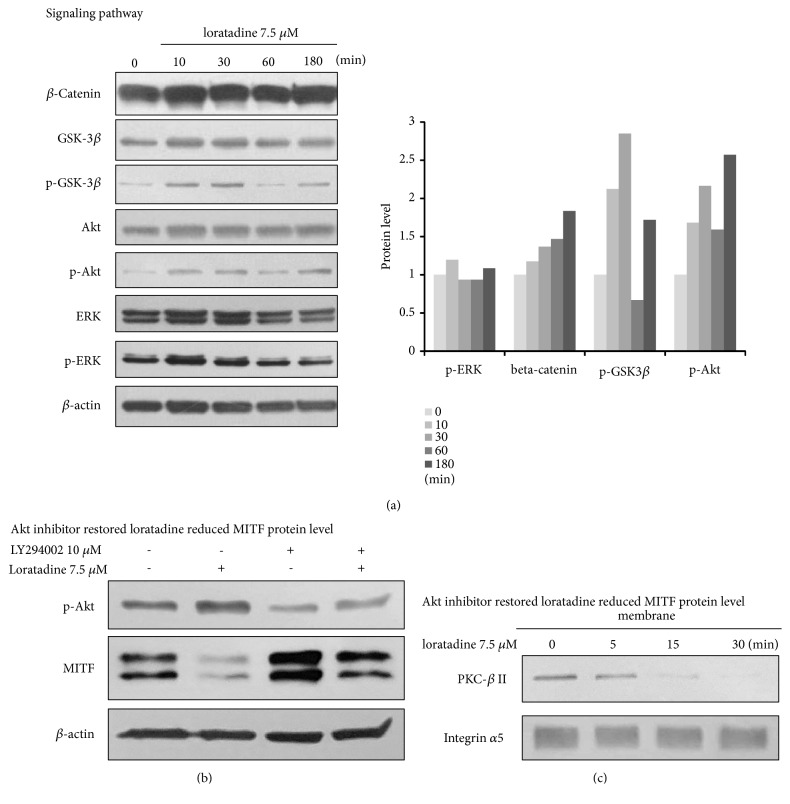
The effects of loratadine on melanogenesis-related signaling pathways. After excluding the influence of PMA by 3 hr starvation, NHM were treated with 7.5 *μ*M loratadine for the indicated times. (a) Whole-cell lysates were analyzed by western blot using antibodies, *β*-catenin, phospho-Erk, phospho-Akt, and phospho-GSK-3*β*, and equal protein loading was confirmed by the *β*-actin levels. Phospho-Akt and phospho-GSK-3*β* were consistently elevated by loratadine. (b) LY294002, a selective inhibitor of PI3K, could reverse the increase of phospho-Akt in loratadine treated NHM. (c) Loratadine treatment reduced the amount of PKC-*β*II in the membrane fraction, thereby decreasing its activity.

To investigate the intracellular signaling pathways, expression levels of *β*-catenin, phospho-GSK-3*β*, phospho-Akt, and phospho-ERK were detected in NHM (Figure 3(a)). To determine the effect of loratadine on PI3K/Akt/GSK-3*β* signaling pathway, phosphorylation of Akt and GSK-3*β* was found to be increased following loratadine treatment, markedly at 30 min. *β*-catenin and phospho-ERK were not altered.

We next evaluated whether LY294002, a selective inhibitor of PI3K, affected the inhibitory effect of loratadine on melanogenesis. As shown in Figure 3(b), loratadine monotreatment significantly reduced the melanin content, whereas cotreatment with LY294002 and loratadine reversely increased the melanin content in NHM. Our results revealed that antimelanogenic effect of loratadine in NHM is associated with activation of PI3K/Akt/GSK-3*β* signaling pathway.

### 3.4. Antimelanogenic Effects of Loratadine Were Associated with Membrane PKC-*β*II in NHM

**Figure 4:**
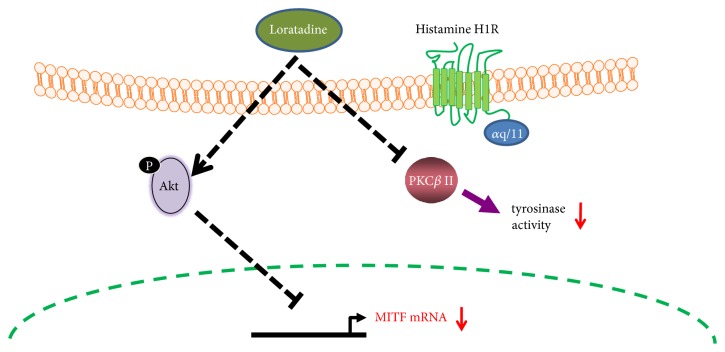
Antimelanogenic effect of H_1_ antihistamine, loratadine via modulating Akt/MITF, and PKC-*β*II signaling.

H1-receptor is one of the G protein-coupled receptors that couples to G*α*_q/11_ proteins, leading to the activation of the calcium/protein kinase C (PKC) pathway. Since, specifically, PKC-*β*II is activator of tyrosinase, we investigated whether loratadine could decrease activity of PKC-*β*II. As shown in Figure 3(c), loratadine treatment reduced the amount of PKC-*β*II in the membrane fraction, thereby decreasing its activity. The molecular mechanisms of antimelanogenic effect of loratadine demonstrated above are summarized in Figure 4.

## 4. Discussion

Histamine acts through 4 different classes of receptors (H_1_, H_2_, H_3_, and H_4_ receptors) on effector cells. Among these 4 receptors, H_1_ and H_2_ receptors have been extensively studied and demonstrated well-known proallergic properties [9]. Since the first synthesized in 1937, H_1_ antihistamines have been some of the most commonly prescribed medicine in dermatologic conditions, including allergic reactions, urticaria, atopic dermatitis, and pruritus. Antihistamines were considered histamine receptor antagonists, but they actually work as inverse agonists by binding to histamine receptors, thereby returning cellular equilibrium and reducing allergic property [10].

Moreover, H_1_ and H_2_ receptors exist on the surface of human melanocytes and melanoma cells [11]. Yoshida et al. [6] have reported that histamine induces melanogenetic effects on human melanocytes by accumulating cyclic adenosine monophosphate (cAMP) and subsequently activating protein kinase A (PKA), especially via the H_2_ receptor. Kim et al. [12] investigated the signaling pathways involved in histamine-induced melanocyte proliferation and melanogenesis. They showed that an H_2_ antihistamine, famotidine, suppressed the effects induced by the H_2_ agonist, amthamine, and histamine itself. Histamine stimulated melanocyte proliferation and melanogenesis via the H_2_ receptor and Erk, CREB, or Akt activation [12]. More recently, it was reported that the H_2_ receptor mediated growth-differentiation factor-15 (GDF-15) could be involved in histamine-induced melanogenesis [13]. In an* in vivo* study, the UVB-induced hyperpigmentation of guinea pig skin was suppressed by topically applying an H_2_ antihistamine [14].

Although the H_1_ receptor is a major therapeutic target of inflammatory skin disorders, there have been few studies about melanogenesis of H_1_ antihistamine [6, 15]. For example, mepyramine, an H_1_ antihistamine, did not inhibit melanogenesis that is induced by histamine [6]. Therefore, first we screened antimelanogenic effects by H_1_ antihistamines using LOPAC chemical library (Table 1). Among them, ebastine, clemisole, terfenadine, and loratadine significantly decreased the melanin content, but loratadine was ultimately selected as its dose-dependent linear hit without affecting cellular viability. Our study found that H_1_ antihistamine, especially loratadine, demonstrates obvious antimelanogenic effects in NHM. Loratadine led to the significant inhibition of mRNA and protein expression level of MITF, which in turn suppressed tyrosinase, a key enzyme that controls melanogenesis.

Akt activation has been reported to reduce melanogenesis via transcriptional downregulation of MITF gene expression [16]. Furthermore, in other mechanism, PI3K/Akt/GSK-3*β* signaling pathway regulates posttranslational modification and proteasomal degradation of MITF protein [17]. In our present study, loratadine suppressed the MITF mRNA expression in NHM, which reversely increased after inhibition of Akt pathway by the selective inhibitor of PI3K, LY294002. Therefore, antimelanogenic effects of loratadine in NHM are shown to be related to activation of PI3K/Akt/GSK-3*β* signaling and the subsequent decrease in the MITF mRNA level.

Unlike H_2_ receptor, which is bound to G_as_ protein and regulates melanogenesis via cAMP/PKA/CREB signaling pathway, H_1_ receptor mainly acts by coupling G_aq/11_ proteins, which in turn activate inositol trisphosphate (IP_3_)/diacylglycerol (DAG) pathway and subsequently localizing PKC enzymes to membrane [9]. PKC-*β*II, a regulator of tyrosinase activity, especially, is known to increase melanogenesis and the activity of PKC-*β*II is determined by the membrane localization [18]. As expected, loratadine did not affect the phosphorylation of CREB, but reduced activity of PKC-*β*II.

Our study had several limitations. Although loratadine showed the antimelanogenic effect at the cellular level, these results do not always provide the same outcomes as a clinical manner. Therefore, for practical application of the results, further clinical studies will be required to determine the therapeutic regimen of loratadine for treatment of hyperpigmentary disorders in humans.

Taken together, we demonstrated strong antimelanogenic effect of H_1_ antihistamine, loratadine via modulating Akt/MITF, and PKC-*β*II signaling. Considering common use of H_1_ antihistamines in dermatologic practice, the antimelanogenic effects of loratadine may have potentially significant and useful roles. Although further clinical studies will be needed, loratadine may give benefits for treatment of hyperpigmentary changes accompanied by various types of dermatitis.

## Figures and Tables

**Table 1:** Summary of the H1 antihistamines screened for antimelanogenesis. Among them, clemisole, ebastine, loratadine, and terfenadine significantly decreased the melanin content (text with *∗*).

Name	Trade name	Therapeutic chemical class	Systematic (IUPAC) name	Molecular weight
First generation antihistamines				

Brompheniramine maleate	Bromfed, Dimetapp, Bromfenex, Dimetane, Lodrane	Alkylamine	3-(4-bromophenyl)-*N*,*N*-dimethyl-3-pyridin-2-yl-propan-1-amine	435.3213
Chlorpheniramine maleate	Chlor-trimeton	Alkylamine	3-(4-chlorophenyl)-*N*,*N*-dimethyl-3-pyridin-2-yl-propan-1-amine	390.8703
Clemastine fumarate	Tavist	Aminoalkyl ethers	(2*R*)-2-{2-[(1*R*)-1-(4-chlorophenyl)-1-phenylethoxy]ethyl}-1-methylpyrrolidine	459.9746
Clemizole hydrochloride^**∗**^	-	-	1-[(4-chlorophenyl)methyl]-2-(pyrrolidin-1-ylmethyl)benzimidazole	362.3053
Diphenhydramine hydrochloride	Benadryl, unisom, sominex, zzzquil	Aminoalkyl ethers	2-(diphenylmethoxy)-*N*,*N*-dimethylethanamine	291.824
Doxylamine succinate	unisom	Aminoalkyl ethers	*(RS*)-*N*,*N*-dimethyl-2-(1-phenyl-1-pyridin-2-yl-ethoxy)-ethanamine	388.4677
Methapyrilene hydrochloride	-	Substituted ethylenediamines	*N*,*N*-dimethyl-*N*′-pyridin-2-yl-*N*′-(2-thienylmethyl)ethane-1,2-diamine	297.8526
Orphenadrine hydrochloride	Norflex, Banflex, Orphenate, Flexoject, Flexon	Aminoalkyl ethers	*N*,*N*-dimethyl-2-[(2-methylphenyl)- phenyl-methoxy]-ethanamine	305.8511
Pheniramine maleate	Avil	Substituted alkylamines	*N*,*N*-dimethyl-3-phenyl-3-pyridin-2-yl-propan-1-amine	356.4253
Promethazine hydrochloride	Phenergan	Phenothiazine derivatives	*(RS*)-*N,N*-dimethyl-1-(10*H*-phenothiazin-10-yl)propan-2-amine	320.8873
Pyrilamine maleate	Pyrlex	Substituted ethylene diamines	*N*-(4-methoxybenzyl)-*N*′,*N*′-dimethyl-*N*-pyridin-2-ylethane-1,2-diamine	401.4664
Triprolidine hydrochloride	Actidil, Myidil, Actifed	other	2-[(*E*)-1-(4-methylphenyl)-3-pyrrolidin-1-yl- prop-1-enyl]pyridine	314.8616

Second generation antihistamines				

Ebastine^**∗**^	Evastin, kestine, ebastel, aleva, ebatrol	Other	4-(4-benzhydryloxy-1-piperidyl)-1-(4-tert-butylphenyl)butan-1-one	469.6731
Cetirizine dihydrochloride	Zirtec, Zyrtec, reactine	Piperazine derivatives	(±)-[2-[4-[(4-chlorophenyl)phenylmethyl]-1- piperazinyl]ethoxy]acetic acid	461.8199
Epinastine hydrochloride	Alesion, Elestat, Purivist, Relestat	other	(*RS*)-3-amino-9,13b-dihydro-1*H*-dibenz(c,f)imidazo(1,5-a)azepine	285.779
Ketotifen fumarate	Zaditor	other	4-(1-Methylpiperidin-4-ylidene)-4,9-dihydro-10*H*-benzo[4,5]cyclohepta[1,2-*b*]thiophen-10-one	425.5075
Loratadine^**∗**^	Claritin, Claratyne	other	Ethyl 4-(8-chloro-5,6-dihydro-11H-benzo[5,6]cyclohepta[1,2-b]pyridin-11-ylidene)-1-piperidinecarboxylate	382.8938
Terfenadine^**∗**^	Seldane, Triludan, Teldane	other	(*RS*)-1-(4-*tert*-butylphenyl)-4-{4-[hydroxy(diphenyl)methyl]piperidin-1-yl}-butan-1-ol	471.6891

Third generation antihistamine				

Fexofenadine hydrochloride	Allegra, Fexidine, Telfast, Fastofen, Tilfur, Vifas, Telfexo, Allerfexo, Flexofen	other	(±)-4-[1-Hydroxy-4-[4-(hydroxydiphenylmethyl)-1-piperidinyl]-butyl]-*α*, *α*-dimethyl benzeneacetic acid	501.6719

## Data Availability

The datasets generated during and/or analyzed during the current study are available from the corresponding author upon reasonable request.

## Supplementary Materials

Supplementary Materials Supplementary Figure 1: effects of H1-receptor agonist and antagonists on melanogenesis. Supplementary Figure 2: effects of loratadine on cellular viability in normal human melanocytes.
