# Supplementary material for: Loratadine, an H1 Antihistamine, Inhibits Melanogenesis in Human Melanocytes
Source: Biomed Res Int. 2019 Mar 17;2019:5971546. doi: 10.1155/2019/5971546 (PMC6441540; doi:10.1155/2019/5971546)
Supplement: Supplementary Materials — Supplementary Figure 1: effects of H1-receptor agonist and antagonists on melanogenesis. Supplementary Figure 2: effects of loratadine on cellular viability in normal human melanocytes. [file 5971546.f1.pdf]

Supplementary Materials

(a)

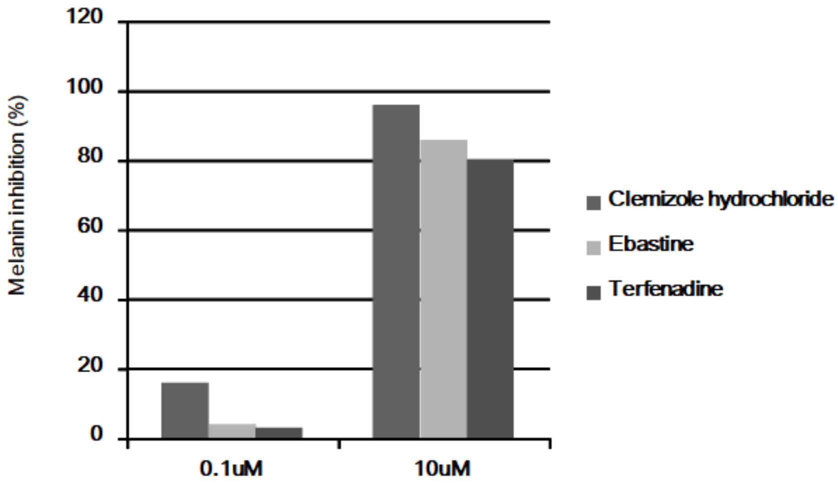

(b)

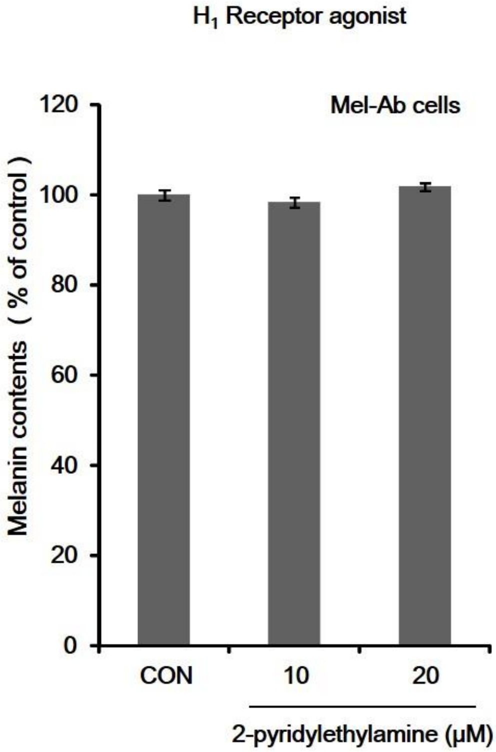

(c)

H1 Receptor agonist

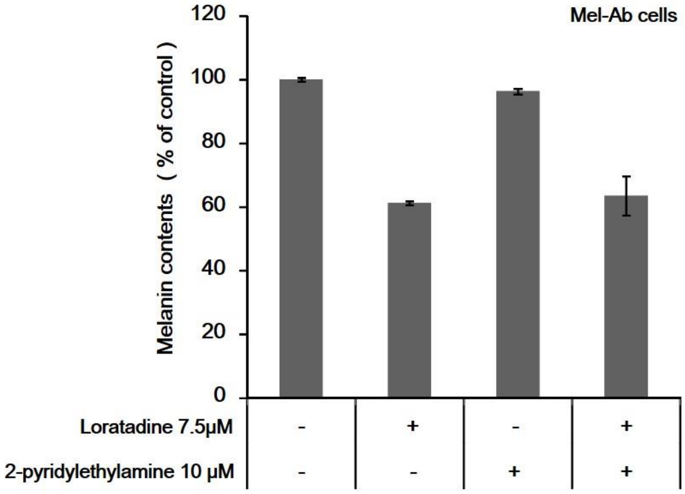

Supplementary Figure 1. Effects of H1-receptor agonist and antagonists on melanogenesis.

(a) B16F10 cells were cultured for 3 days in the presence of various H1-receptor antagonists including clemizole, ebastine and terfenadine, and the melanin content was measured. (b) Mel-ab cells were cultured for 3 days with 10-20 μM 2- pyridylethylamine, a H1-receptor agonist. The melanin content was not affected by 2- pyridylethylamine treatment. (c) 2-pyridylethylamine did not reverse the melanin content which were decreased by loratadine.

(a)

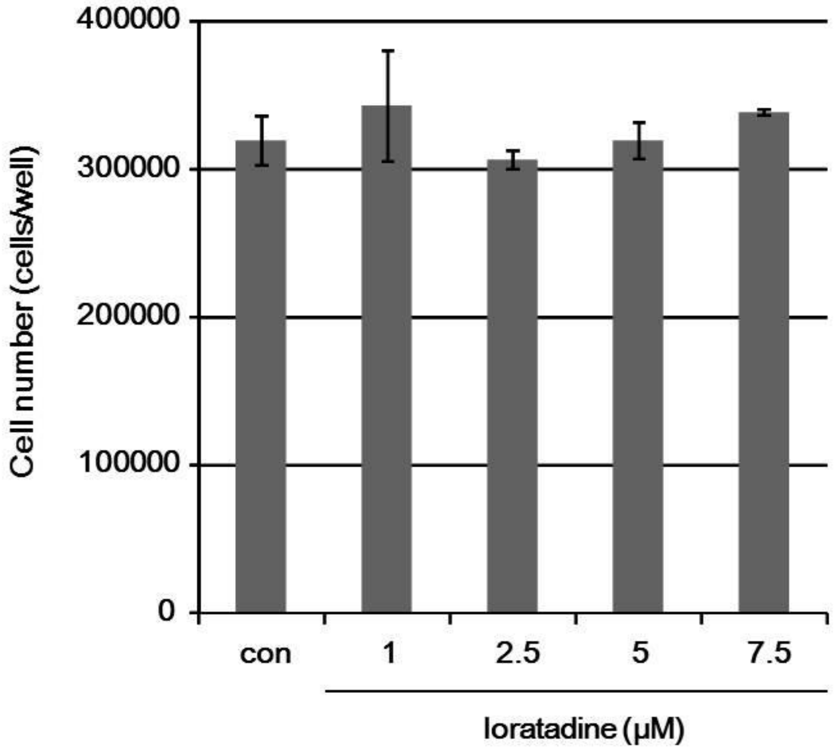

(b)

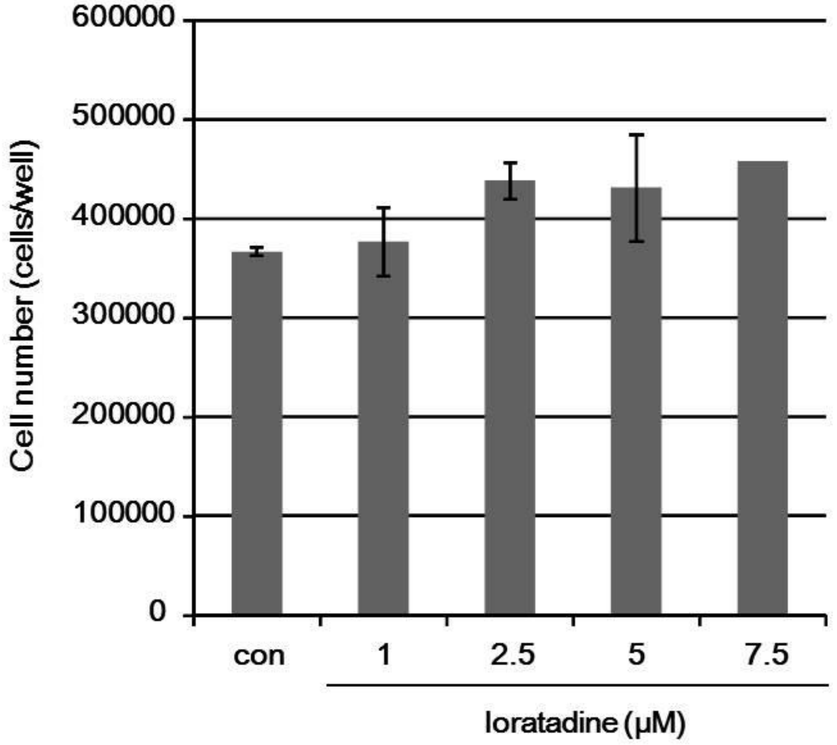

Supplementary Figure 2. Effects of loratadine on cellular viability in normal human

melanocytes (NHM).

NHM were cultured with 1.0 - 7.5 μM loratadine for (a) 3 days and (b) 5 days. The cell number of NHM was determined using trypan blue counting assay. Cell viability was not affected by treatment with 1.0 - 7.5 μM loratadine for 3 days and 5 days.
